# Effect of hydroxychloroquine in patients with IgA nephropathy with insufficient responses to immunosuppressive therapy: a retrospective case-control stud*y*

**DOI:** 10.1186/s12882-020-02141-9

**Published:** 2020-11-10

**Authors:** Chen Tang, Ji-Cheng Lv, Su-Fang Shi, Yu-Qing Chen, Li-Jun Liu, Hong Zhang

**Affiliations:** Renal Division, Peking University First Hospital, Peking University Institute of Nephrology, Key Laboratory of Renal Disease, Ministry of Health of China, Key Laboratory of Chronic Kidney Disease Prevention and Treatment (Peking University), Ministry of Education, Beijing, 100034 People’s Republic of China

**Keywords:** IgA nephropathy, Hydroxychloroquine, Immunosuppressive therapy, Proteinuria

## Abstract

**Background:**

Hydroxychloroquine (HCQ) is a well-known immunomodulator that was recently used in immunoglobulin A (IgA) nephropathy (IgAN) due to its antiproteinuric effects. We investigated the effects of HCQ in patients with IgAN whose proteinuria remained above 1 g/d after conventional immunosuppressive (IS) therapy.

**Methods:**

This study was a retrospective case-control study. Twenty-six patients with IgAN who received HCQ and had insufficient responses to IS therapy (corticosteroid (CS) therapy with/without IS agents) were included. Twenty-six matched historical controls who received conventional IS therapy were selected using propensity score matching. The clinical data from 6 months were compared.

**Results:**

Proteinuria at baseline was comparable between the “IS therapy plus HCQ” and “conventional IS therapy” groups (2.35 [interquartile range (IQR), 1.47, 2.98] vs. 2.35 [IQR, 1.54, 2.98] g/d, *p* = 0.920). A significant reduction in proteinuria was noted in IgAN patients with HCQ treatment (2.35 [IQR, 1.47, 2.98] vs. 1.10 [IQR, 0.85, 1.61] g/d, *p* = 0.002). The percent reduction in proteinuria at 6 months was similar between the two groups (− 39.81% [− 66.26, − 12.37] vs. -31.99% [− 67.08, − 9.14], *p* = 0.968). The cumulative frequency of patients with a 50% reduction in proteinuria during the study was also comparable between the two groups (53.8% vs. 57.7%, *p* = 0.780). No serious adverse events (SAEs) were observed during the study.

**Conclusions:**

Use of HCQ achieved has similar reduction in proteinuria compared to conventional IS therapy in patients with IgAN who had insufficient responses to IS therapy.

**Supplementary Information:**

The online version contains supplementary material available at 10.1186/s12882-020-02141-9.

## Background

Immunoglobulin A (IgA) nephropathy (IgAN) is the most common primary glomerular disease worldwide, and it is a leading cause of end-stage kidney disease (ESKD) [1]. IgAN is a complex disease with variable clinical and pathological features and a partially understood pathogenesis. Therefore, no specific therapies targeting the key pathways involved in its pathogenesis are available [2]. Under these circumstances, blood pressure optimization and renin-angiotensin-aldosterone system inhibitors (RAASis) are the primary management methods [3]. The 2012 Kidney Disease Improving Global Outcomes (KDIGO) Clinical Practice Guidelines for glomerulonephritis suggest that patients with persistent proteinuria (≥1 g/d and an estimated glomerular filtration rate [eGFR] > 50 ml/min per 1.73 m^2^) after 3–6 months of optimized supportive care should receive a 6-month course of corticosteroid (CS) therapy [4]. However, the effect of CS in IgAN is controversial. Although the STOP-IgAN and TESTING studies showed that treatment with CS in IgAN led to a reduction in proteinuria, treatment with CS was associated with a significantly increased risk of serious adverse events (SAEs) [5]. Several clinical trials examined whether combination therapy with steroids and immunosuppressive (IS) agents, such as cyclophosphamide (CTX) [6], mycophenolate mofetil [7], tacrolimus [8], and leflunomide [9], produced better renal outcomes. However, the results were inconclusive [10].

Hydroxychloroquine (HCQ) is a widely used medication for the treatment of rheumatoid arthritis and systemic lupus erythematosus, and a number of experimental and clinical observations confirmed its beneficial effects [11]. Our previous studies showed that the application of HCQ in patients with IgAN effectively reduced proteinuria. Compared with conventional RAASi treatment alone, HCQ more effectively reduced proteinuria after 6 months [12, 13]. Compared with CS therapy, HCQ was only slightly less effective for reducing proteinuria and had fewer side effects [14]. It may be effective in treating IgAN through suppressing Toll-like receptor (TLR) stimulation and reduced production of cytokines [15]. Therefore, HCQ might be an alternative or supplement to CS therapy for patients with IgAN who have insufficient responses to conventional IS therapy. Therefore, in this study, we aimed to compare the efficacy and safety of HCQ to those of conventional IS therapy in such patients.

## Methods

### Study design and study population

This study was a retrospective case-control study performed at a single centre. We retrospectively searched the medical records from an IgAN database at Peking University First Hospital. This database contained 1360 patients from 1994 to 2019. The inclusion criteria were patients with IgAN who had insufficient responses to IS therapy (CS therapy with/without IS agents) and were treated with HCQ. Insufficient response to IS therapy in IgAN patients was defined as persistent proteinuria above 1 g/d after at least 3 months of IS therapy. The exclusion criteria were a lack of baseline or follow-up data, less than 3 months of HCQ therapy, and the use of other IS agents within 3 months before or after HCQ therapy.

**Fig. 1:**
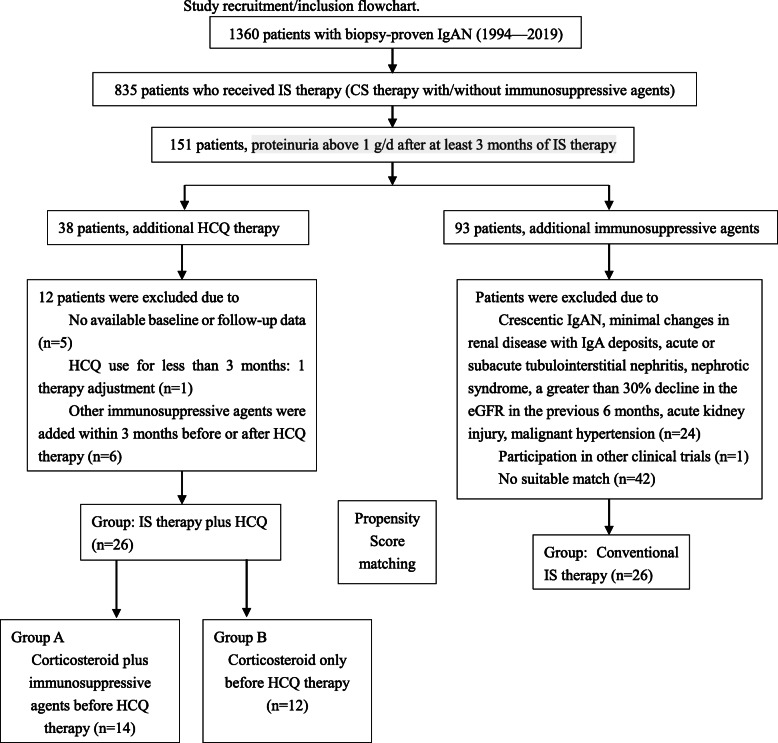
Study recruitment/inclusion flowchart

For each patient who received HCQ therapy, we selected matched controls from patients who received conventional IS agents in combination with CS therapy using propensity score matching based on age, sex, eGFR and proteinuria levels. A 1:1 fashion control group with the closest propensity score (within 0.2 standard deviations) for each HCQ user was selected, excluding HCQ users without a suitable match and the rest of controls. The number of matches determined the size of sample. Patients with crescentic IgAN (defined by crescents in greater than 50% of glomeruli), minimal renal disease changes with IgA deposits, acute or subacute tubulointerstitial nephritis, nephrotic syndrome (proteinuria level ≥ 3.5 g/d and serum albumin ≤30 g/l), a greater than 30% decline in eGFR in the previous 6 months, acute kidney injury, and malignant hypertension were excluded from both groups [14] (Fig. 1).

### Interventions

The HCQ dose varied according to the baseline eGFR. The dose was 0.2 g twice daily for patients with an eGFR greater than 60 ml/min/1.73 m^2^, and the dose was 0.1 g two or three times daily for patients with an eGFR between 30 and 60 ml/min/1.73 m^2^. However, the dose was 0.1 g once daily for patients with an eGFR between 15 and 30 ml/min/1.73 m^2^ [13]. All patients were given sufficient angiotensin-converting enzyme inhibitors (ACEIs)/angiotensin receptor blockers (ARBs) for at least 3 months. The dose of RAASis was titrated to ensure that the patients were receiving the maximum allowable or tolerated dose, and blood pressure control was optimized according to the KDIGO guidelines for IgAN. Additional antihypertensive medications were prescribed if the blood pressure was > 130/80 mmHg with RAASi treatment alone.

CS treatment included oral steroids (0.8–1.0 mg/kg/d, maximum 60 mg/d) for 2 months. This therapy was reduced by 5 mg every 2 weeks and stopped within 6 to 8 months. The other was intravenous steroid (methylprednisolone (500 mg) for 3 days at 1, 3 and 5 months followed by prednisone (15 mg/d p.o.) for 6 months) [14].

Other IS agent doses were standard doses based on patient renal function and physician experience. The IS agents used included CTX, mycophenolate mofetil, cyclosporine A (CsA), leflunomide and FK506.

Observation lasted for 6 months or was stopped until termination of HCQ treatment or the addition of other IS agents.

### Data collection and outcome measures

Baseline characteristics were reviewed for the following data: age, gender, Oxford Classification MESTC score, the duration of illness to the initiation of IS therapy, concomitant use of an RAASi, 24-h total urinary protein excretion, serum creatinine level (SCr), and eGFR. The eGFR was calculated using the Chronic Kidney Disease Epidemiology Collaboration (CKD-EPI) formula and SCr. These data were collected every 1–2 months during the follow-up. All assays were performed at a laboratory in the Department of Clinical Laboratory of Peking First Hospital using standard methods.

The primary outcome was defined as a change in proteinuria from baseline to 6 months. The secondary outcomes were defined as the percent changes in proteinuria from baseline to 2 and 4 months, the cumulative frequency of patients with a 50% decrease in proteinuria, a change in eGFR during the follow-up and adverse events (AEs) [14].

SAEs were defined as any medical events that met one or more of the following criteria: resulted in death, was life-threatening, required inpatient hospitalization or prolongation of an existing hospitalization, resulted in persistent or significant disability, severe infection requiring hospitalization, osteonecrosis or bone fracture, gastrointestinal haemorrhage or perforation, new-onset diabetes mellitus, new-onset cataract or fundus lesions, severe liver dysfunction or allergies requiring hospitalization, and major cardiocerebral vascular disease (including fatal/nonfatal myocardial infarction, fatal/nonfatal stroke, and heart failure) [16].

AEs were collected from medical records. Patients receiving HCQ treatment were referred to an ophthalmologist for retinal evaluations every 3–6 months.

### Statistical analysis

Normally distributed data are presented as the means ± SD, and non-normally distributed data are presented as medians (Q25, Q75). Categorical data are summarized as counts and percentages. Clinical parameters during the follow-up period were compared using Student’s t-tests (for normally distributed, continuous variables), Wilcoxon signed-rank tests (for nonnormally distributed, continuous variables) or χ2 tests (for nominal variables), as appropriate. The cumulative frequency of patients with a 50% decrease in proteinuria was estimated using the Kaplan-Meier method, and time represented the period from baseline to the first occurrence of a 50% decrease in proteinuria. Univariable regression followed by multivariable logistic regression was performed to determine the independent predictors of a proteinuria reduction of at least 50%.

All missing information was treated as missing data without imputation. All analyses were performed using SPSS Statistics, version 22.0 (SPSS Inc., Chicago, IL, USA). A *p*-value less than 0.05 was considered statistically significant.

## Results

### Baseline characteristics

A total of 26 eligible patients with IgAN from 1994 to Dec 2019 were included in this study. All patients received HCQ therapy after at least 3 months of CS therapy with/without IS agents, and no patients were treated with other immunosuppressants after receiving HCQ therapy. On average, they received IS therapy for 7.4 (4.9, 10.8) months before taking HCQ. Two of the 26 patients stopped CS use when HCQ treatment was initiated, and the remaining patients continued to use CS plus HCQ for 3.38 (1.81, 7.64) months.

Among the 26 patients treated with HCQ, 14 patients were initially treated with CS therapy plus IS agents before HCQ treatment (group A), half of whom were treated with CS plus CTX, and the average cumulative dose of cyclophosphamide was greater than 7 g. All 14 patients in this group stopped taking IS agents when starting HCQ treatment. The other 12 patients were initially treated with CS therapy alone before HCQ treatment (group B).

Twenty-six patients with IgAN treated with conventional IS therapy were selected using propensity score matching (Fig. 1). They were initially treated with CS therapy for 3.9 (2.1, 11.3) months then treated with additional IS agents because of an insufficient response to CS therapy alone. Fourteen of these patients were treated with CTX.

The baseline characteristics of all patients are shown in Table 1. The characteristics of groups A and B are shown in Additional file 1. The baseline proteinuria levels (2.35 [1.47, 2.98] vs. 2.35 [1.54, 2.98] g/d, *p* = 0.920) and eGFR (47.65 [34.65, 67.48] vs. 51.59 [26.47, 82.32] ml/min/1.73 m2, *p* = 0.855) were comparable between the IS therapy plus HCQ and conventional IS therapy groups.

**Table 1:** Main clinical and laboratory characteristics at baseline in patients with IgAN

	IS therapy plus HCQ *N* = 26	Conventional IS therapy *N* = 26	*P*-value
Gender			
Male/female	9/17	10/16	0.773
Age at renal biopsy (years)	28.8 ± 10.2	30.8 ± 12.2	0.522
Duration of IS therapy before HCQ/other IS agents (months)	7.4 (4.9,10.8)	3.9 (2.1,11.3)	0.079
Proteinuria level before IS therapy (g/d)	2.52 (1.66,5.60)	2.27 (1.54,4.36)	0.727
Baseline proteinuria (g/d)	2.35 (1.47,2.98)	2.35 (1.54,2.98)	0.920
Baseline eGFR (ml/min/1.73 m^2^)	47.65 (34.65,67.48)	51.59 (26.47,82.32)	0.855
Oxford Classification			
M 0/1	2/21	9/17	0.030
E 0/1	11/12	14/12	0.674
S 0/1	7/16	15/11	0.056
T 0/1/2	3/18/2	11/11/4	0.034
C 0/1/2	5/15/3	10/16/0	0.104
RAASi therapy	25	22	
ACEI alone	14	4	
ARB alone	11	6	
ACEI plus ARB	0	12	
Use of statins	6	8	
Corticosteroid treatment			
Corticosteroid pulse therapy	13	8	
Oral corticosteroids	13	18	
Immunosuppressive agents	14/26	26/26	
Cyclophosphamide	6	14	
Mycophenolate mofetil	3	3	
Cyclosporine A	2	4	
Leflunomide	2	4	
FK506	1	1	

The histological scores of 3 patients in the group “IS therapy plus HCQ” were unavailable because they underwent renal biopsy in other clinics

### Primary outcome

**Fig. 2:**
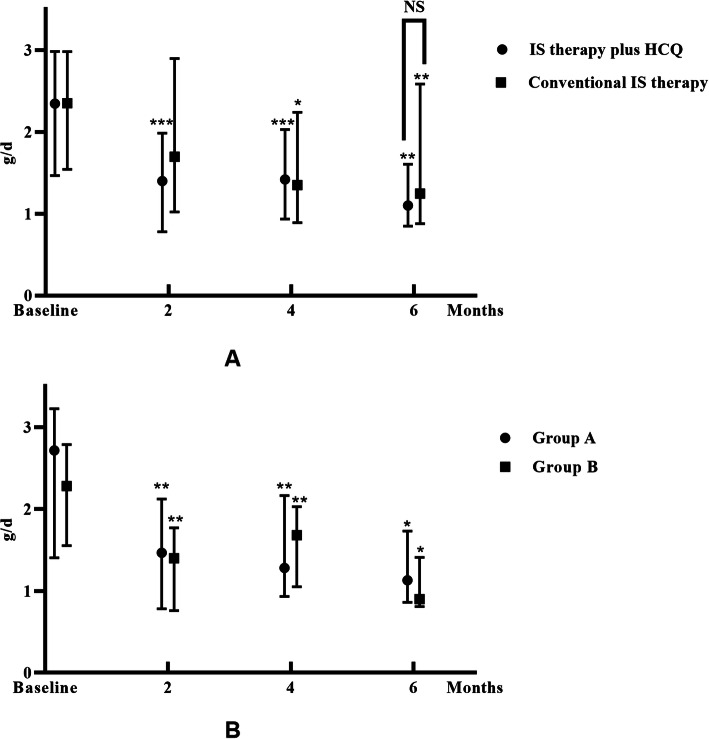
Urinary protein excretion of all patients during the follow-up period. The dots represent the median proteinuria values, and the bars represent the 25th and 75th percentiles. The level of UTP was compared to the baseline level every 2 months. * *p* < 0.05; ** *p* < 0.01; *** *p* < 0.001. **a**. The groups “IS therapy plus HCQ” and “conventional IS therapy”. **b**. Groups A and B. Group A: Patients treated with corticosteroid plus immunosuppressive agents before HCQ therapy. Group B: Patients treated with corticosteroid only before HCQ therapy

The level of proteinuria at 6 months decreased from 2.35 [1.47, 2.98] g/d to 1.10 [0.85, 1.61] g/d (*p* = 0.002) in the IS therapy plus HCQ group and from 2.35 [1.54, 2.98] g/d to 1.24 [0.87, 2.58] g/d (*p* = 0.009) in the conventional IS therapy group. No significant difference in proteinuria at 6 months was identified between the two groups (*p* = 0.312). For groups A and B, the level of proteinuria decreased from 2.72 [1.41, 3.23] g/d to 1.13 [0.86, 1.73] g/d (*p* = 0.022) and from 2.28 [1.55, 2.78] g/d to 0.90 [0.81, 1.41] g/d (*p* = 0.043), respectively (Fig. 2).

**Fig. 3:**
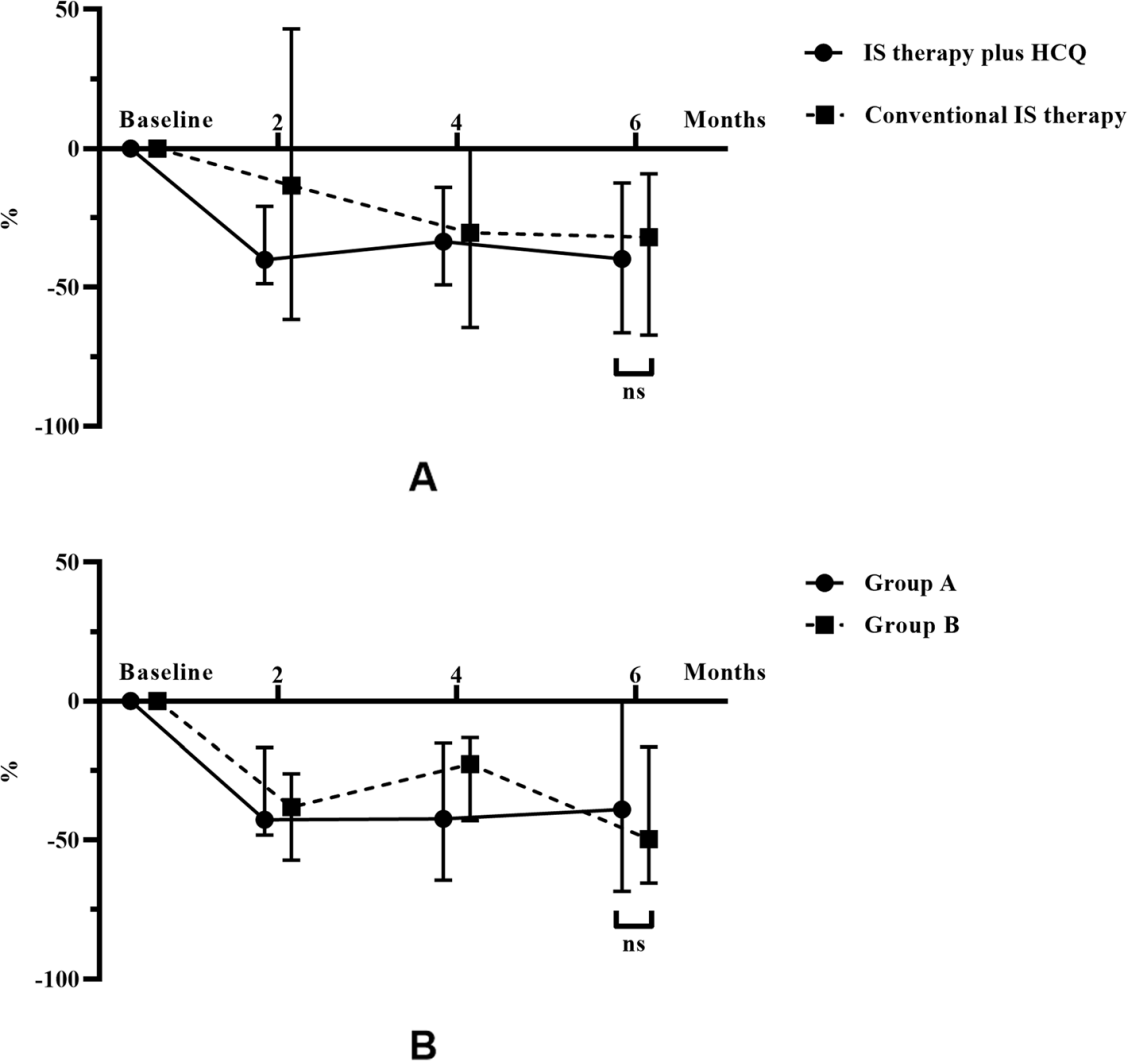
Changes in urinary protein excretion of all patients during the follow-up period. The dots represent the median percentage changes in proteinuria, and the bars represent the 25th and 75th percentiles. The change rate was compared between two groups at 6 months. * *p* < 0.05; ** *p* < 0.01; *** *p* < 0.001. **a**. The groups “IS therapy plus HCQ” and “conventional IS therapy”. **b**. Groups A and B. Group A: Patients treated with corticosteroid plus immunosuppressive agents before HCQ therapy. Group B: Patients treated with corticosteroid only before HCQ therapy

The percent reductions in proteinuria from baseline to 6 months were − 39.81% [− 66.26, − 12.37] in the IS therapy plus HCQ group and − 31.99% [− 67.08, − 9.14] in the conventional IS therapy group. No significant difference in the percent change was found between the two groups (*p* = 0.968). For groups A and B, the change rates were − 38.93% [− 68.50, 0.00] and − 44.72% [− 63.30, − 19.57], respectively. No significant difference in proteinuria at 6 months was observed between groups A and B (*p* = 0.898) (Fig. 3).

### Secondary outcomes

The percent reductions in proteinuria from baseline to 2 months were − 40.17% [− 48.71, − 20.88] for patients treated with HCQ and − 13.4% [− 61.66,42.97] for patients treated with conventional IS therapy (*p* = 0.347). The percent reductions in proteinuria from baseline to 4 months were − 33.62% [− 49.15, − 13.95] for patients treated with HCQ and − 30.43% [− 64.4, 0.00] for patients treated with conventional IS therapy (*p* = 0.768) (Fig. 3).

**Fig. 4:**
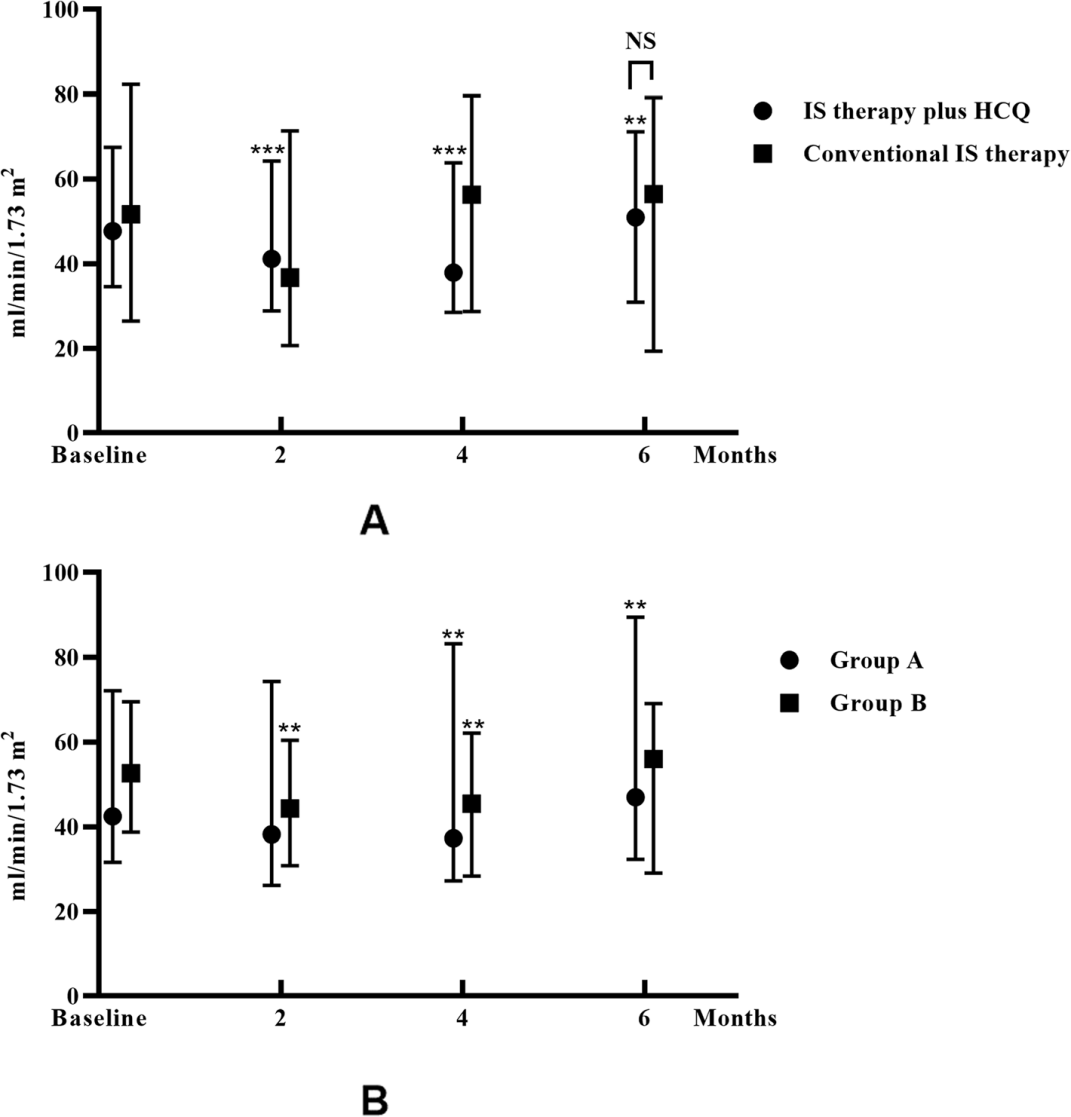
The eGFR levels of all patients during the follow-up period. The dots represent the median values, and the bars represent the 25th and 75th percentiles. The eGFR was compared to the baseline eGFR every 2 months. * *p* < 0.05; ** *p* < 0.01; *** *p* < 0.001. **a**. The groups “IS therapy plus HCQ” and “conventional IS therapy”. **b**. Groups A and B. Group A: Patients treated with corticosteroid plus immunosuppressive agents before HCQ therapy. Group B: Patients treated with corticosteroid only before HCQ therapy

During the follow-up, the eGFR remained roughly stable. For patients in the IS therapy plus HCQ group, the eGFR was 47.65 [34.65, 67.48] ml/min/1.73 m^2^ before treatment with HCQ. After 6 months of HCQ therapy, the eGFR was 50.91 [30.96, 71.14] ml/min/1.73 m^2^, which was slightly higher than the beginning of treatment (*p* = 0.005). For patients treated with conventional IS therapy, no significant change in the eGFR was noted (51.59 [26.47, 82.32] vs. 56.39 [19.37, 79.24] ml/min/1.73 m^2^, *p* = 0.305). No significant difference in the eGFR at 6 months was found between the two groups (50.91 [30.96, 71.14] vs. 51.59 [26.47, 82.32] ml/min/1.73 m^2^, *p* = 0.821) (Fig. 4).

**Fig. 5:**
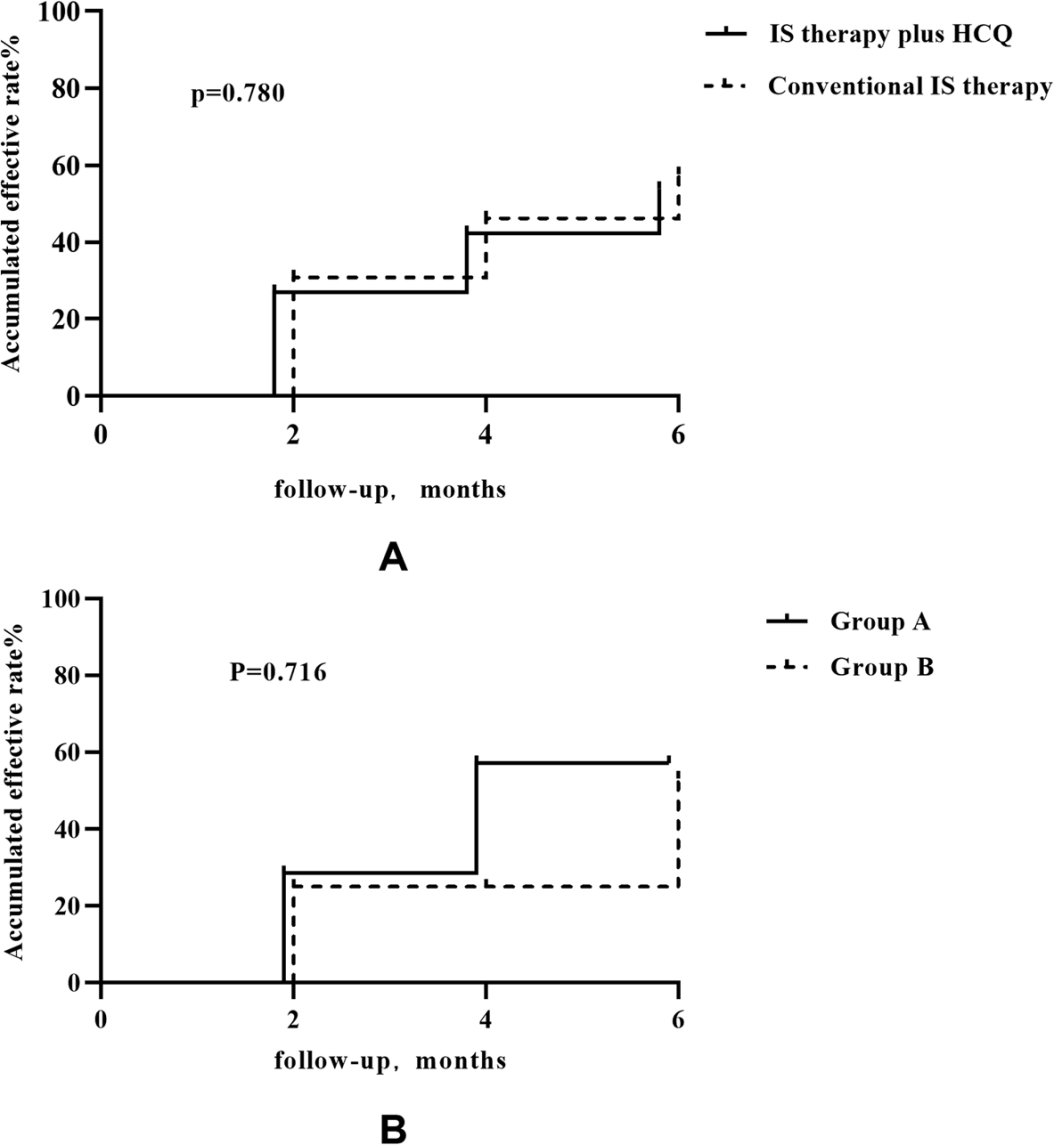
The cumulative frequency of a 50% reduction in proteinuria during the follow-up period. **a**. The accumulated effective rate for the two groups “IS therapy plus HCQ” and “conventional IS therapy”. **b**. The accumulated effective rate for each group in the “IS therapy plus HCQ” group. Group A: Patients treated with corticosteroid plus immunosuppressive agents before HCQ therapy. Group B: Patients treated with corticosteroid only before HCQ therapy

The cumulative frequencies of patients with a 50% decrease in proteinuria at 6 months were 53.8% for patients treated with HCQ and 57.7% for patients treated with conventional IS therapy. No significant difference (*p* = 0.780) was observed between the two groups (Fig. 5).

Multivariable analysis with logistic regression did not show a specific predictive factor for a greater effective proteinuria reduction during therapy (Table 2).

**Table 2:** Clinical and histological characteristics influencing the effective proteinuria reduction frequency (a reduction in proteinuria of at least 50%) using univariate and multivariate logistic regression

	Univariate			Multivariate		
Characteristic	OR	95%CI	*P* value	OR	95%CI	*P* value
Age (years)	1.001	(0.952,1.053)	0.961	1.028	(0.947,1.115)	0.511
Sex (male/female)	2.308	(0.747,7.128)	0.146	1.475	(0.272,8.000)	0.652
Proteinuria level before IS therapy (g/d)	1.024	(0.813,1.290)	0.838	0.883	(0.580,1.344)	0.562
Baseline eGFR (ml/min/1.73 m^2^)	1.009	(0.990,1.028)	0.357	0.988	(0.960,1.017)	0.415
Baseline proteinuria (g/d)	1.561	(0.815,2.992)	0.180	2.175	(0.845,3.597)	0.107
Oxford Classification						
M 0/1	0.514	(0.129,2.052)	0.346	0.206	(0.020,2.092)	0.182
E 0/1	0.665	(0.216,2.050)	0.477	0.478	(0.071,3.207)	0.394
S 0/1	0.124	(0.034,0.452)	0.232	0.049	(0.007,0.322)	0.486
T 0/1/2	0.525	(0.203,1.359)	0.184	0.680	(0.104,4.458)	0.688
C 0/1/2	0.657	(0.230,1.876)	0.433	1.039	(0.209,5.164)	0.962
Optimal RAASi therapy	0.259	(0.025,2.675)	0.259	0.255	(0.020,3.198)	0.290
HCQ therapy	1.364	(0.457,4.071)	0.578	0.478	(0.071,3.207)	0.447
Conventional IS therapy	0.733	(0.246,2.189)	0.578	0.795	(0.252,2.509)	0.696

### Safety and AEs

No SAEs were recorded during the 6 months of observation. One patient in group A stopped HCQ therapy because of nausea and diarrhoea, and one patient had skin pigmentation without changes in medications. No AEs were recorded in group B. One patient in the conventional IS therapy group stopped CsA because of papules, and one patient stopped CTX because of pregnancy.

## Discussion

The current study investigated the efficacy of HCQ after IS therapy for proteinuria in patients with IgAN and found a similar effect as conventional IS therapy in matched historical controls. All of the patients with IgAN underwent medication adjustments because of insufficient responses to initial IS or CS therapy. The protein excretion of these patients remained above 1 g/d after at least 3 months of IS therapy (CS therapy with/without IS agents). After these adjustments, the proteinuria levels in both groups decreased further at 6 months, and no significant difference in the percent change in proteinuria was noted between the two groups. HCQ may be effective in combination with other immunosuppressants for slowing disease progression, which suggests that HCQ is an alternative or supplement for patients with IgAN who have an insufficient response to IS therapy in the future.

Previous studies confirmed the antiproteinuric and immunomodulatory effects of HCQ in patients with IgAN in a short follow-up period, and patients showed good tolerance and compliance with HCQ therapy [13]. However, these studies focused on the effect of HCQ as an additional agent to RAASis and excluded patients who had recently taken CS or IS agents [12, 14]. Compared to previous studies, the current study focused on patients whose protein excretion could not be maintained below 1 g/d even with adequate IS treatment. We found that most patients started HCQ near the end stage of IS therapy, which may guarantee that HCQ effectively reduced proteinuria and minimized interference from pretreatment immunosuppression as much as possible. The patients in the current study initiated IS therapy approximately 2.9 (0.1, 27.2) months after kidney biopsy, which also increased the connection between the pathology and clinical manifestations of the disease. The patients treated with HCQ in this study had higher percentages of T1 and T2 disease on pathology than the patients treated with conventional IS therapy (86.9% vs. 57.7%, *p* = 0.034), which generally predicted lower renal survival and a higher risk of progression to end-stage renal disease (ESRD) [17]. However, the results suggested that HCQ may be effective in patients with IgAN with relatively severe pathology even after IS therapy.

Aberrant glycosylation of O-linked glycans in the hinge region of IgA1 plays a key role in the pathogenesis of IgAN [18]. However, the mechanism driving the effect of HCQ in reducing proteinuria and alleviating the disease in patients with IgAN was not clarified. HCQ has a considerable anti-inflammatory effect [19]. Patients in the HCQ group had proportionally higher M and E scores, which generally reflects glomerular inflammation [17]. IS therapy before HCQ treatment seemed effective in this study because proteinuria decreased to some degree, and the anti-inflammatory effect may have played a role. However, inflammatory factors are not the main cause of IgAN because the effect of IS therapy is not clear [20]. Recent studies suggested that TLR-mediated innate immune responses may play a role in IgAN progression. An overexpression of TLR4, TLR7, TLR8 and TLR9 is detected in patients with IgAN, which may be related to disease severity and activity [21]. HCQ blocks TLRs, such as TLRs 3, 7, 8, and 9, to prevent immune system activation [19]. Complement activation by IgA1-containing immune complexes is involved in the development and progression of IgA nephropathy [22], and relatively lower levels of C3 are associated with a greater risk of disease progression [23]. HCQ inhibited complement fixation and activation and prevented complement from splitting in vivo and in vitro [24]. These results may explain how HCQ decreased proteinuria in patients with IgAN.

The lag effects in the reduction in proteinuria in response to steroid or IS agents are noteworthy in the current study. Of the 26 patients treated with HCQ, 14 patients were treated with steroids plus IS agents (group A), and 12 patients were treated with steroids alone before HCQ therapy (group B). We did not stop immunotherapy completely for a period of time before adding HCQ, but with the observed trend in proteinuria, the IS therapy at that time would likely not provide further benefits (Additional file 2). All patients in group A stopped IS agents when taking HCQ, and most of them simply maintained a small dose of CS. According to the results of the TESTING study, the effect of steroids on the reduction in proteinuria was more prominent in the first 3 months then gradually waned in the next 3 months [25]. The patients enrolled in our study received IS therapy for 7.4 (4.9, 10.8) months on average before taking HCQ, which gave us more confidence that HCQ played an important role in the reduction in proteinuria during the follow-up. We also observed a relatively rapid decrease in proteinuria within 2 months after the addition of HCQ, which may be a result of the combination of CS and HCQ because most of the patients maintained low doses of CS for approximately 3 months. This speculation must be confirmed in future studies.

Multivariable analysis with logistic regression did not show a specific predictive factor for a greater effective proteinuria reduction during therapy, which may be limited by the sample size. However, HCQ is effective in combination with other immunosuppressants for slowing the deterioration of kidney function in the context of partial remission. For patients with IgAN who have insufficient responses to conventional IS therapy, HCQ may be used as an alternative or a supplement to CS therapy, rather than other additional IS agents, to minimize the side effects of CS and other IS agents.

Our study had several limitations. First, as a single-centre retrospective study, selection bias of patients receiving steroids with or without immunosuppression was inevitable. In addition, this was a small-scale study with a short follow-up period, which may have led to some variability and bias. We didn’t previously regard HCQ as an alternative treatment for patients with IgAN. In such cases, CS combined with IS treatment constituted the most suitable strategy. The long-term effect and safety of HCQ alone or HCQ plus CS in patients with IgA nephropathy and insufficient responses to IS therapy are not known. Multicentre clinical studies and mechanistic research are needed to be done in the future.

## Conclusions

Use of HCQ achieved has similar reduction in proteinuria compared to conventional IS therapy in patients with IgAN who had insufficient responses to IS therapy. It suggests that HCQ may be an alternative treatment or a supplement for IgAN in the future to reduce the side effects of IS agents.

## Supplementary Information

**Additional file 1: Table S1.** Main clinical and laboratory characteristics at baseline in patients treated with HCQ.

**Additional file 2: Figure S1.** Changes in urinary protein excretion and eGFR levels of all patients before initiation of HCQ therapy. The dots represent the median values, and the bars represent the 25th and 75th percentiles.

## Data Availability

The datasets used and/or analysed during the current study are available from the corresponding author upon reasonable request.

## Abbreviations

HCQ: Hydroxychloroquine
IgAN: Immunoglobulin A nephropathy
ESKD: End-stage kidney disease
RAASi: Renin-angiotensin-aldosterone system inhibitor
CS therapy: Corticosteroid therapy
IS therapy: Immunosuppressive therapy
ACEI: Angiotensin-converting enzyme inhibitor
ARB: Angiotensin receptor blocker
CTX: Cyclophosphamide
MMF: Mycophenolate mofetil
CsA: Cyclosporine A
SAEs: Serious adverse events
AEs: Adverse events
SCr: Serum creatinine
eGFR: Estimated glomerular filtration rate (calculated using the CKD-EPI equation)
