# Supplementary material for: Effect of hydroxychloroquine in patients with IgA nephropathy with insufficient responses to immunosuppressive therapy: a retrospective case-control study
Source: BMC Nephrol. 2020 Nov 10;21:469. doi: 10.1186/s12882-020-02141-9 (PMC7653892; doi:10.1186/s12882-020-02141-9)
Supplement: Supplementary file 1 — Additional file 1: Table S1. Main clinical and laboratory characteristics at baseline in patients treated with HCQ. [file 12882_2020_2141_MOESM1_ESM.docx]

**Table 1.** Main clinical and laboratory characteristics at baseline in the patients treated with HCQ

|  | Group A (N=14) | Group B (N=12) |
| --- | --- | --- |
| Gender |  |  |
| Male/female | 6/8 | 3/9 |
| Age at renal biopsy (years) | 26.1±12.1 | 32.3±6.0 |
| Duration of IS therapy before HCQ/other immunosuppressive agents (months) | 8.1(5.8,14.5) | 5.8(3.5,9.0) |
| Duration of overlap of CS and HCQ (months) | 3.9(2.8,8.9) | 3.2(1.1,7.5) |
| Baseline proteinuria (g/d) | 2.72(1.41,3.23) | 2.28(1.55,2.78) |
| Baseline eGFR (ml/min/1.73 m2) | 42.42(31.65,72.13) | 52.59(38.70,69.55) |
| M 0/1 | 2/11 | 0/10 |
| E 0/1 | 6/7 | 5/5 |
| S 0/1 | 5/8 | 2/8 |
| T 0/1/2 | 2/10/1 | 1/8/1 |
| C 0/1/2 | 4/7/2 | 1/8/1 |
| RAASi therapy | 13 | 12 |
| ACEI alone | 5 | 9 |
| ARB alone | 8 | 3 |
| ACEI plus ARB | 0 | 0 |
| Use of statins | 3 | 3 |
| Corticosteroid treatment |  |  |
| Corticosteroid pulse therapy | 5 | 8 |
| Oral corticosteroids | 9 | 4 |
| Immunosuppressive agents (% of patients) | 100% | 0% |
| Cyclophosphamide | 6 |  |
| Mycophenolate mofetil | 3 |  |
| Cyclosporine A | 2 |  |
| Leflunomide | 2 |  |
| FK506 | 1 |  |

The histological scores of one patient in Group A and two patients in Group B were unavailable because they underwent renal biopsy in other clinics.
