# Supplementary material for: Effect of hydroxychloroquine in patients with IgA nephropathy with insufficient responses to immunosuppressive therapy: a retrospective case-control study
Source: BMC Nephrol. 2020 Nov 10;21:469. doi: 10.1186/s12882-020-02141-9 (PMC7653892; doi:10.1186/s12882-020-02141-9)
Supplement: Supplementary file 2 — Additional file 2: Figure S1. Changes in urinary protein excretion and eGFR levels of all patients before initiation of HCQ therapy. The dots represent the median values, and the bars represent the 25th and 75th percentiles. [file 12882_2020_2141_MOESM2_ESM.docx]

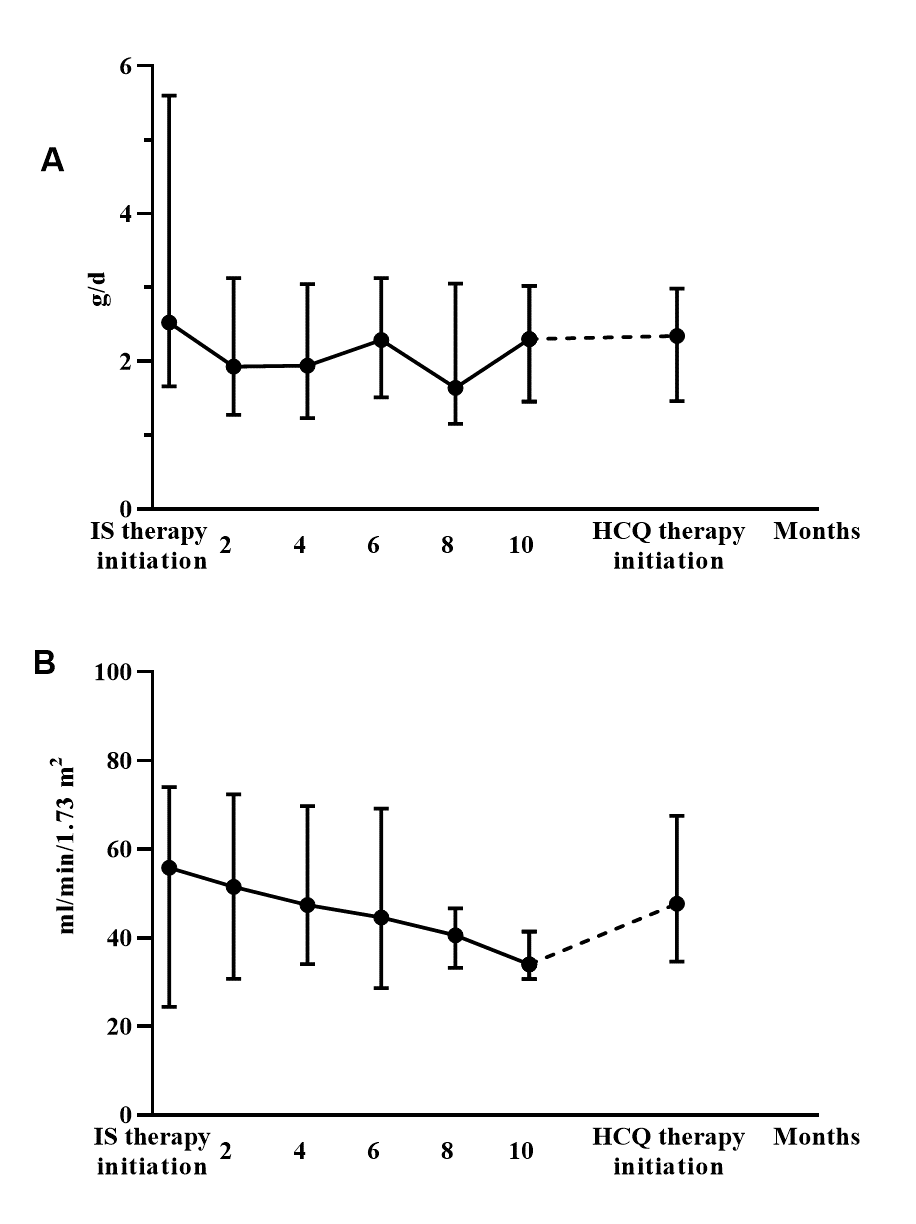


Additional file 2.

**Figure 1.** Changes in urinary protein excretion and eGFR levels of the enrolled patients before initiation of HCQ therapy. The dots represent the median value, and the bars represent the 25th and 75th percentiles.

1. Changes in the urinary protein excretion levels of the enrolled patients before initiation of HCQ therapy
2. Change in the eGFR levels of the enrolled patients before initiation of HCQ therapy
